# Enchondroma in the Fibular Diaphysis: A Case Report

**DOI:** 10.7759/cureus.67210

**Published:** 2024-08-19

**Authors:** Mihai Aurel Costache, Sergiu Iordache, Adrian Cursaru, Mihnea Popa, Bogdan Serban, Bogdan Cretu, Andreea Marinescu, Catalin Cirstoiu

**Affiliations:** 1 Orthopaedics and Traumatology, University Emergency Hospital, Bucharest, ROU; 2 Radiology and Imaging, University Emergency Hospital, Bucharest, ROU

**Keywords:** treatment choices, enchondroma, fibula fracture, resection, bone tumors

## Abstract

Chondroma is a benign tumor formation that occurs through the proliferation of cartilaginous tissue. It can be located centrally (enchondroma) or peripherally, often appears between 10-30 years of age, and is commonly discovered incidentally. This case report describes a 46-year-old woman presenting with pain in the left calf and partial functional impotence. The onset of her symptoms was affirmatively marked by a mild skiing-related trauma. Following protocol, X-ray imaging (antero-posterior and lateral views) of the calf was performed, with the only finding being a solitary bony lesion, with internal calcifications, sclerotic margin and radiolucent internal matrix. Facing the uncertainty of diagnosis from a clinical and radiographic standpoint, it was decided to admit the patient for further evaluation and start the standard protocol of imaging investigations - computed tomography, magnetic resonance imaging, and bone scintigraphy - and determine the subsequent therapeutic behavior. Differential diagnosis between enchondroma and low-grade chondrosarcoma can be difficult due to their histopathological similarity. The therapy of choice in enchondroma comprises non-surgical treatment (observation) if the lesion remains unaltered in imaging, or curettage/filling with bone substitutes/allografts, but considering the advanced age and interruption of the bone cortex in our case, we opted for curative surgical treatment.

## Introduction

Bone tumor can be defined as a new growth of tissue that is characterized by uncontrolled growth of abnormal cells. Bone tumors represent an important chapter in musculoskeletal pathology. The most common benign tumors that occur in the locomotor system are chondroma, osteoid osteoma, giant cell tumor, aneurysmal cyst, and fibroblastic dysplasia [1].

Chondroma is a benign tumor formation that occurs through the proliferation of cartilaginous tissue. It can be located centrally (enchondroma) or peripherally. Enchondroma represents 13% of benign bone tumor cases, which are frequently located at the metaphyses of long bones. Among long bones, chondroma commonly occurs in the metaphyseal regions, especially those of the proximal humerus and distal femur, and less commonly in the proximal tibia (0.7%) or fibula (0.2%), or in flat ostea such as the pelvic bone and ribcage, leading to challenges in diagnosis [2].

Usually, enchondroma is asymptomatic and discovered incidentally during a routine check-up, with an average age of appearance of 10-30 years [3-5]. The process of diagnosing bone tumors relies on clinical aspects and proper imaging techniques, where differentiating between benign or malignant tumor formation is established on the basis of anatomopathological and immunohistochemical examination. This differentiation is relevant in the context of initiating the right course of treatment.

## Case presentation

A 46-year-old woman presented to our Emergency Orthopedics & Trauma Room with pain in her left calf and partial functional impotence. The onset of her symptoms was affirmatively marked by a mild skiing-related trauma. Clinical-anamnestic examination indicated a spontaneous debut of the pain resulting from blunt injury to the lateral side of the left calf, which gradually intensified during physical exercise. Conservatory treatment with non-steroidal anti-inflammatory drugs (NSAIDs) did not successfully alleviate the symptoms. Local examination failed to reveal positive Celsian signs or bruising.

**Figure 1:**
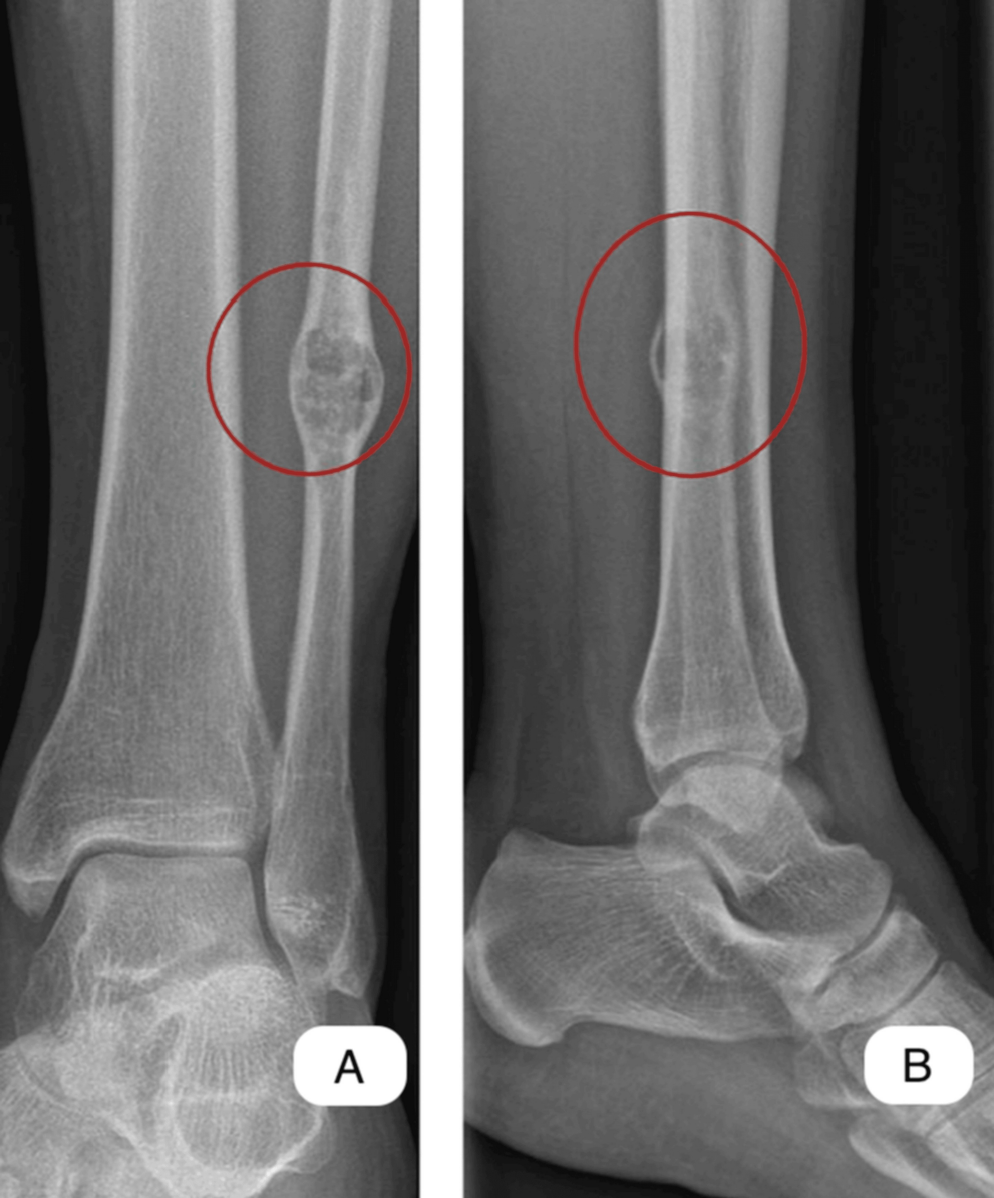
X-ray view of the distal 1/3 of the fibula shaft A - antero-posterior view B - lateral (profile) view

Following protocol, X-rays (antero-posterior and lateral views) of the calf were performed, with the only finding being a solitary bony lesion measuring approximately 12/14 mm in the coronal plane located in the distal 1/3 fibula shaft (Figure 1). The lesion has an eccentric location, a well-defined sclerotic margin and the internal matrix appears predominantly radiolucent, with possible internal calcifications. Also, there is mild cortical thinning over the lesion, but no evidence of cortical breakthrough or periosteal reaction on X-ray which suggests the lesion is likely slow-growing; the surrounding soft tissues appear unremarkable, with no visible soft tissue mass or edema. Considering the appearance of the tumor formation on X-ray, the differential diagnosis at this stage is made between enchondroma, non-ossifying fibroma, fibrous dysplasia and of course chondrosarcoma.

Upon admission to our department, from a biological point of view, the patient did not show changes in blood count (Table 1).

**Table 1:** Laboratory values of the patient at baseline

Biochemical Data	Patient’s Value	Normal Range
WBC (white blood cells)	9.3	3.8–11.8 × 10^3^/μL
HGB (hemoglobin)	12.9	10.9–14.3 g/dL
PLT (platelets)	345	179–408 × 10^3^/μL
Fibrinogen	315	238–498 mg/dL
ALKP (alkaline phosphatase)	54	40–136 U/L
Fe (iron)	110	50–160 mg/dL
CRP (C-reactive protein)	0.4	0–5 mg/L
Breast tumor marker CA 15-3	11.3	0-31.3 U/mL
Ovarian tumor marker CA 125	13.5	0–35 U/mL
Gastric tumor marker CA 19-9	5.4	0–35 U/mL

**Figure 2:**
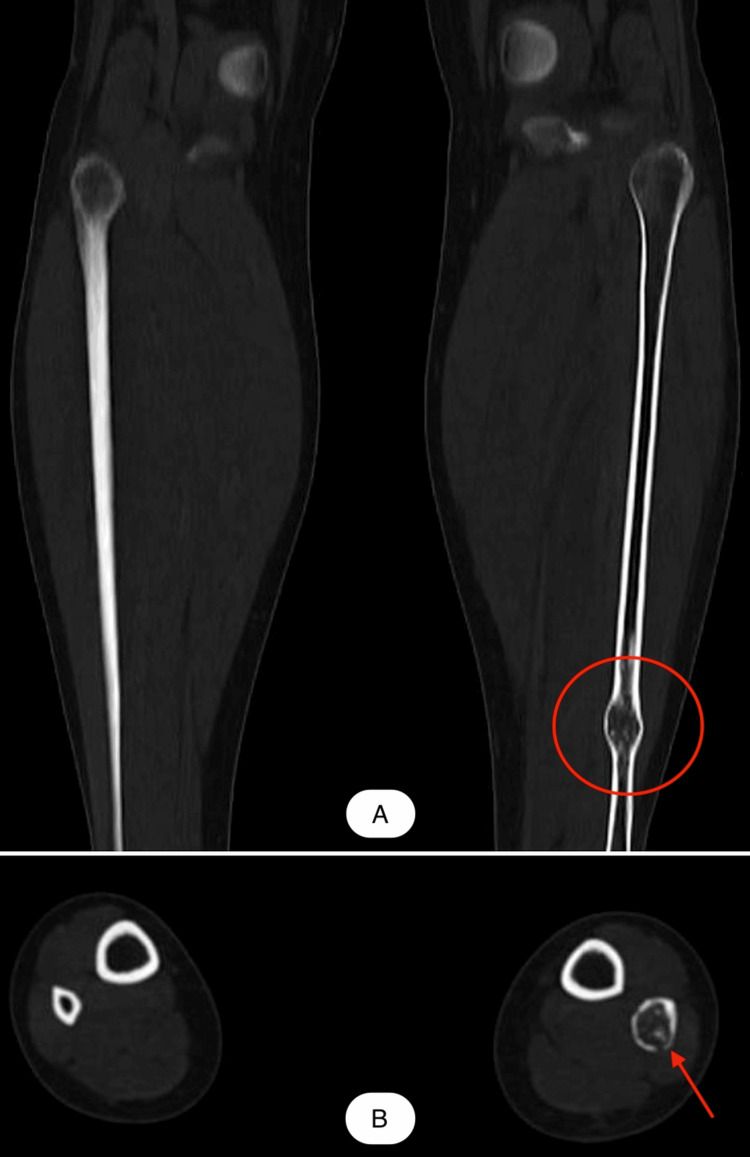
Computed tomography (CT) scan distal 1/3 of the fibula shaft before surgery A - coronal view B - axial view

Facing the uncertainty of diagnosis from a clinical and radiographic standpoint, we aimed to rule out a low-grade chondrosarcoma. Consequently, computed tomography (CT) scans of the thorax, abdomen, and pelvis (TAP), and the left calf were performed. No occult malignancies were identified in the TAP regions; in contrast, on the shaft of the left fibula, we were able to localize an osseous lesion that increased the diameter of the distal diaphysis, with dimensions of 12-14 mm transaxially, extending over a length of approximately 22 mm, which caused the thinning of the bone cortex and presented a fracture path involving the postero-lateral cortex, without displacement or changes in the adjacent soft tissues (Figure 2).

**Figure 3:**
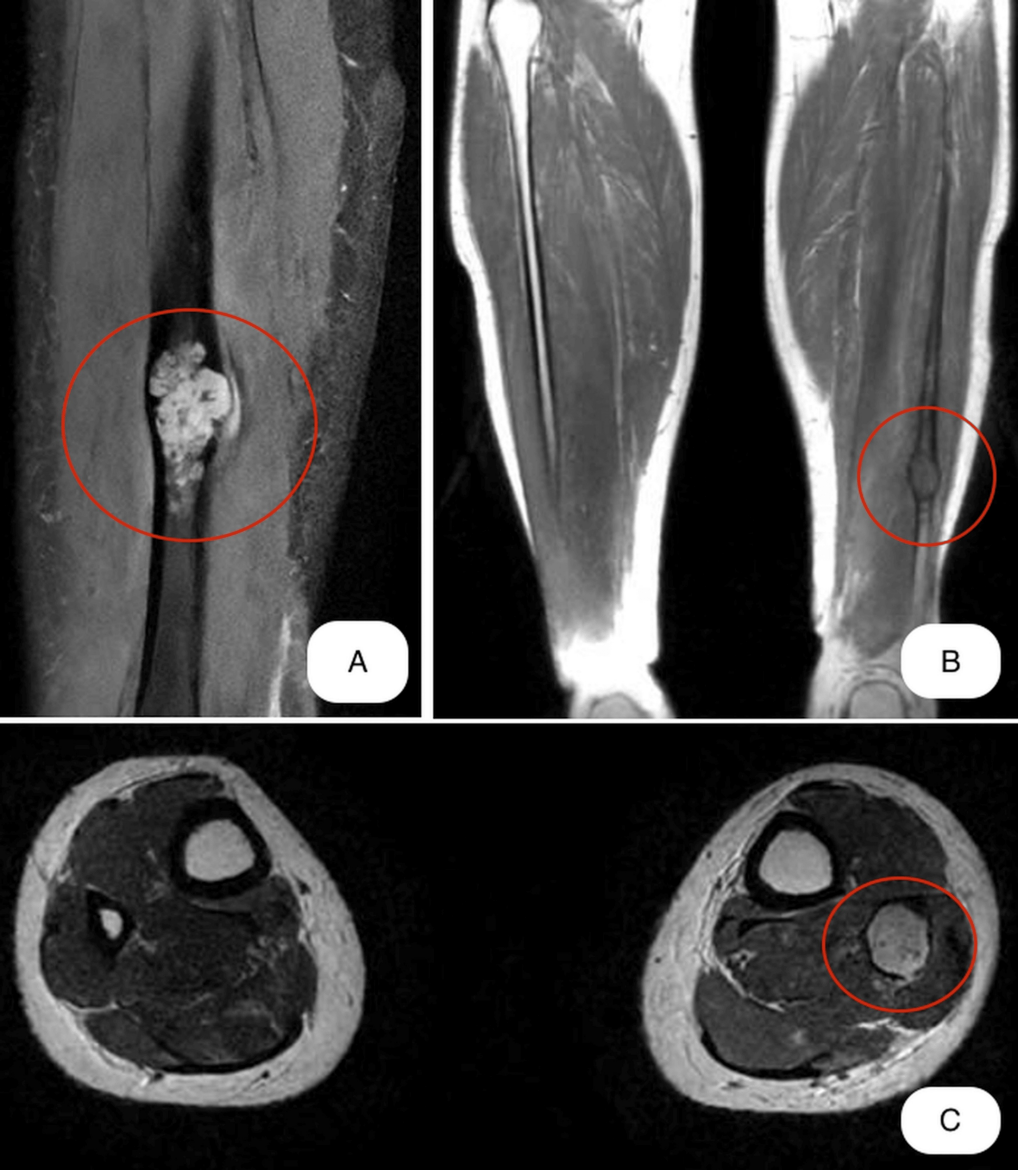
Magnetic resonance imaging (MRI) distal 1/3 of the fibula shaft before surgery A - sagittal view B - coronal view C - axial view

Magnetic resonance imaging (MRI) was performed at the level of the calf. It revealed an intraosseous osteolytic lesion centered at the level of the medullary canal. The lesion thinned the posterior cortical bone, which was highlighted in the inferior third of the fibula. Moreover, a discontinuity of cortex was observed at the level of the lesion which measured approximately 29 mm in the cranio-caudal plane and 14-16 mm in the axial plane. Intramedullary millimeter nodular lesions were also observed inside the tumor formation in the distal 1/3 of the fibula shaft (Figure 3).

**Figure 4:**
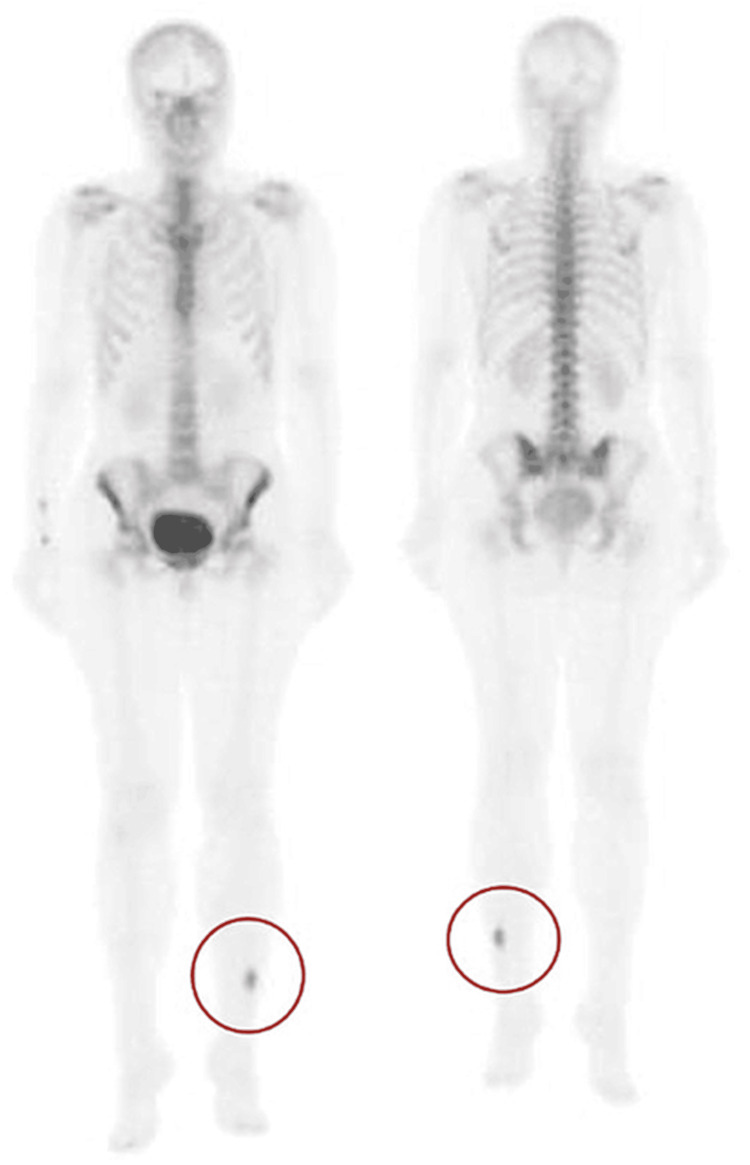
Bone scintigraphy (whole body scan) highlighting the bone lesion in the left fibula

To further analyze the characteristics of the lesion, a whole-body bone scintigraphy was performed. An intensely metabolically active osseous process at the level of the inferior third of the left fibula was identified, visible in all phases (Figure 4).

Managing potential malignancies of the musculoskeletal system is a challenge that demands rigorous preoperative strategies. The choice of surgical approach is of utmost importance, especially when considering biopsies, as the surgeon ought to minimize the further spreading of the tumor cells and concomitantly determine the proper level of resection with oncological margins.

**Figure 5:**
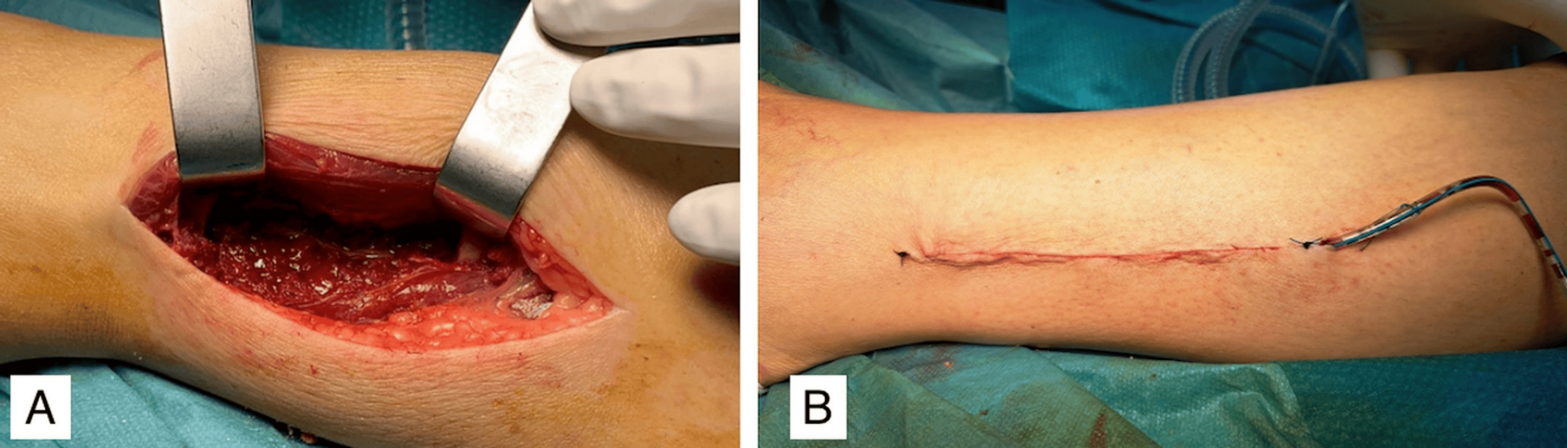
Intraoperative image A - resection area of the tumor B - intradermal suture

**Figure 6:**
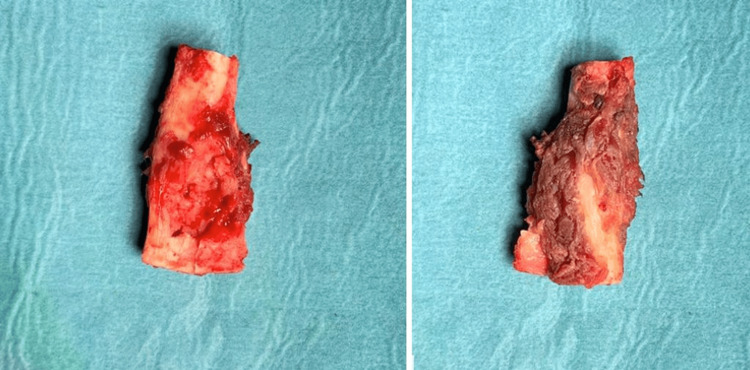
Macroscopic image of the resected bone lesion

In the next step, we carried out an excisional biopsy/resection of the fibular lesion within oncological limits, accessing the inferior third of the peroneal region directly via a lateral incision (Figures 5, 6).

**Figure 7:**
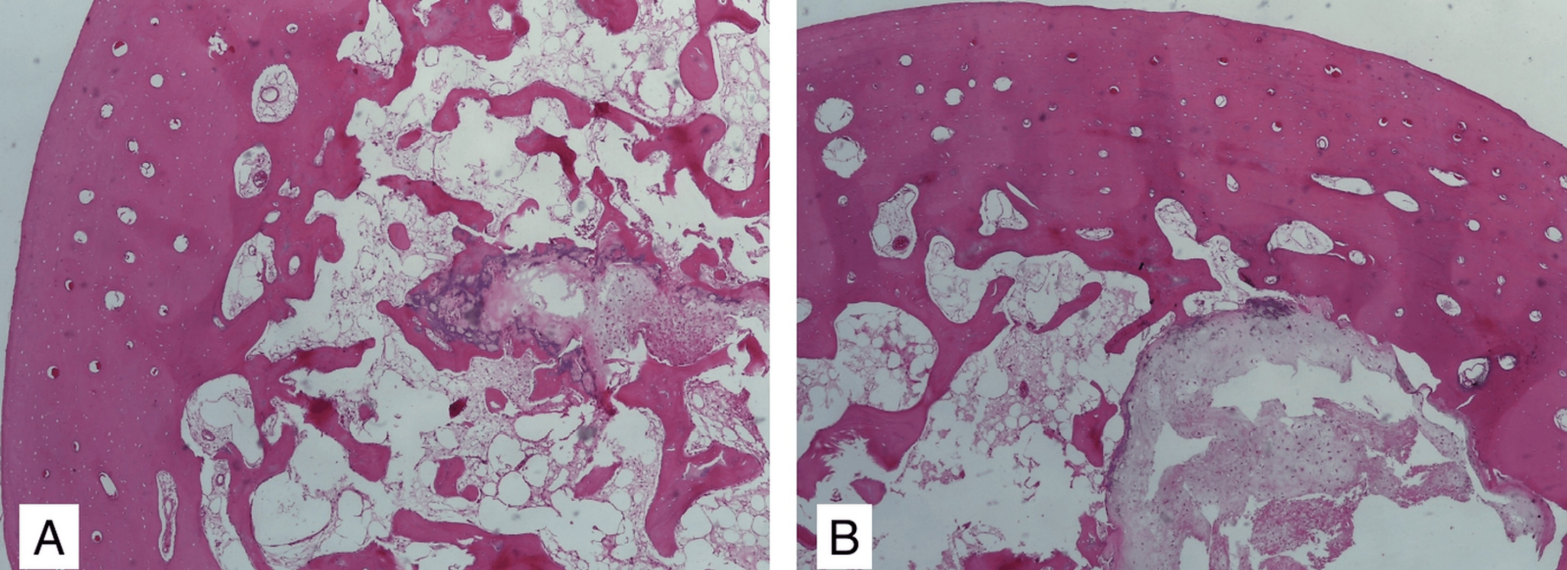
Histopathological image A - Intramedullary enchondromatous lesion consisting of hyaline cartilage with a slightly lobulated interlamellar arrangement B - hypocellular intramedullary chondroid tumor proliferation formed by basophilic matrix and chondroblasts organized in dyads, with the bone cortex unaffected

The resection piece of bone was sent for histopathological examination, in which proliferations of chondrocytes grouped in lobules with low cell density, and a relatively monomorphic and slightly hyperchromic nucleus were identified, arguing for the diagnosis of benign tumor formation, more precisely enchondroma. In addition, resection limits were sent, this one being without tumor invasion (Figure 7).

At discharge, the patient was prescribed an anticoagulant, anodynes, and NSAIDs. She underwent specialized post-surgical recovery and care in a clinic designated for such patients. After only two weeks, her symptoms significantly diminished. Given the psychological aftermath of such an affliction, it was recommended that the patient be included in a psycho-medical rehabilitation program.

**Figure 8:**
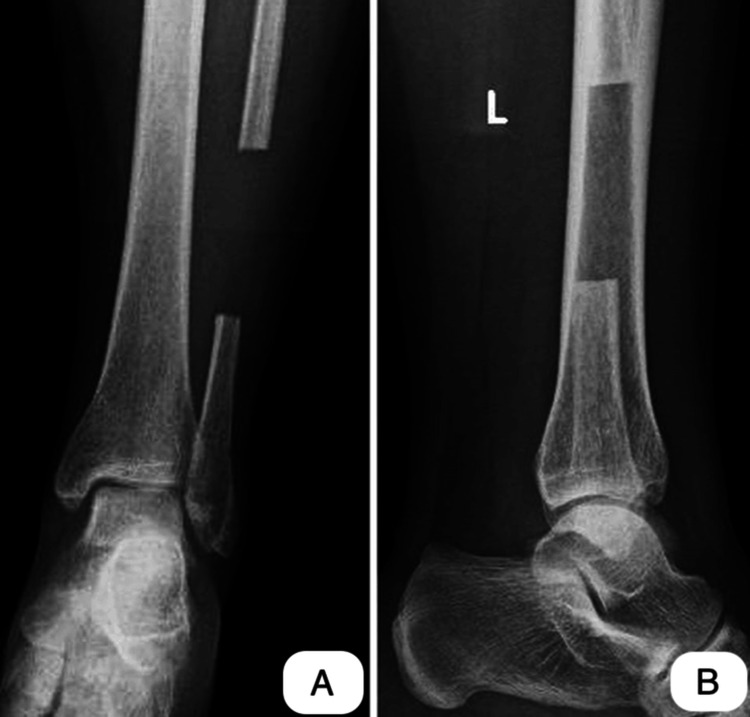
X-ray view after surgery A - antero-posterior view B - lateral view

**Figure 9:**
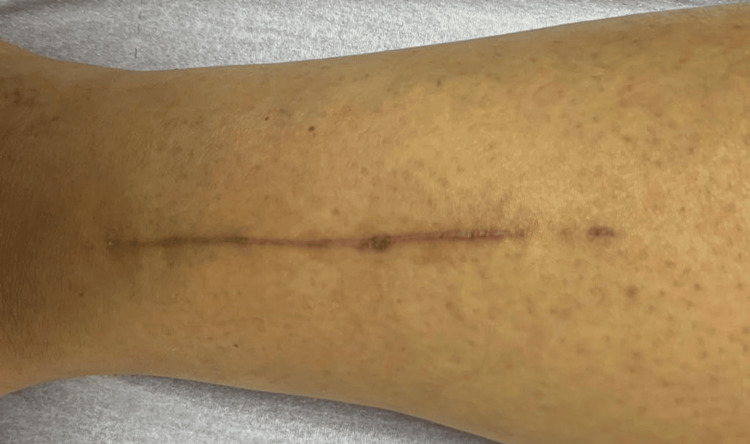
Post-operative image at the 45-day control

At a 45-day post-operative check-up, the patient fully supported herself on the left calf with no complaints (Figures 8, 9).

The patient was recommended to continue the recovery program and come for periodic reassessment.

## Discussion

Enchondroma is routinely identified solely by its radiological features alone. According to the World Health Organization, the diagnostic criteria for enchondroma are as follows: presence of cartilaginous matrix, absence of cortical invasion, absence of extension in the soft parts, and absence of cellular atypia [6-7]. A differential diagnosis is made with low-grade chondrosarcoma, and bone infarction [8]. Treatment can be non-surgical or surgical. The non-surgical and most common treatment in the case of enchondroma is follow-up through periodic imaging investigations, whereas surgical treatment consists of curettage-filling with allografts/bone substitute or resection.

The main purpose of this study was to establish management guidance in the case of enchondroma. The WHO (World Health Organization) in 2013 renamed low-grade chondrosarcoma as “atypical cartilaginous tumor” [8].

The potential for an enchondroma to progress into a secondary chondrosarcoma is a well-established fact. Clinically, the occurrence of non-mechanical pain or night pain at any age warrants concern and immediate further investigation. In fact, pain is typically the symptom that suggests a benign lesion may have become malignant. However, benign enchondromas can also cause pain, meaning that pain does not rule out the possibility of a benign enchondroma. Conversely, the absence of pain does not entirely exclude the diagnosis of chondrosarcoma [9]. Also, if the lesion is radiologically inactive and the patient doesn’t have any pain, a biopsy is not necessary. If the patient develops symptoms, the standard imaging investigation protocol for neoplastic lesions should be followed: CT scan of the TAP region plus the affected segment, scintigraphy of the entire skeleton, and MRI if necessary [9-10].

Distinguishing an enchondroma from a low-grade chondrosarcoma is challenging due to their histopathological similarity. Most enchondromas are small, between 1-3 cm, whereas a chondrosarcoma is larger, approximately 5 cm. If one or more mitotic figures are identified, it is highly indicative of malignancy [10-12]. Sustained pain associated with radiologically identified discontinuity of the bone cortex and invasion of the surrounding soft tissues further support the hypothesis of a malignancy [13-15]. However, the incidence of transformation is less in small bones (hand, feet) and is higher for the shoulder and pelvis. In solitary enchondromas, the risk for malignant transformation is less than 1%, while in multiple enchondromatosis the risk is between 5-25% [16,17].

Surgical treatment remains a topic of debate in patients diagnosed with enchondroma/atypical cartilaginous tumor: resection of the tumor with wide surgical margins or intralesional curettage which is also considered as adequate treatment according to the World Health Organisation [18,19].

Leerapun et al., in a study involving 70 patients, reported 13 cases treated with intralesional curettage and 57 cases with segmental resection. They observed one local recurrence in each group and found no difference in survival between the two treatment methods [20].

In our case, the bone lesion was resected within oncological limits, and the specimens were sent for histopathological examination. Timely intervention and regular follow-up resulted in a good outcome, and there were no signs of recurrence six moths post-surgery.

## Conclusions

Enchondroma is a cartilage-originating benign tumor that is infrequently found in patients over 40 years old, with differential diagnosis being made with a malignant tumor formation, or chondrosarcoma. These tumors can be problematic when they become aggressive or occur in unusual locations when it comes to the choice of treatment. Solitary enchondromas that grow aggressively and become symptomatic should be treated surgically. The therapy of choice in enchondroma comprises non-surgical treatment (observation) if the lesion remains unaltered in imaging, or curettage/filling with bone substitutes/allografts, but considering the advanced age and interruption of the bone cortex in our case, we opted for curative surgical treatment.
